# The History, Present and Future of Allergen Standardization in the United States and Europe

**DOI:** 10.3389/fimmu.2021.725831

**Published:** 2021-09-14

**Authors:** Julia Zimmer, Jennifer Bridgewater, Fatima Ferreira, Ronald van Ree, Ronald L. Rabin, Stefan Vieths

**Affiliations:** ^1^Division of Allergology, Paul-Ehrlich-Institut, Langen, Germany; ^2^Division of Bacterial, Parasitic, and Allergenic Products, Office of Vaccines Research and Review, Center for Biologics Evaluation and Research, US Food and Drug Administration, Silver Spring, MD, United States; ^3^Department of Biosciences, Paris Lodron University of Salzburg, Salzburg, Austria; ^4^Department of Experimental Immunology and Department of Otorhinolaryngology, Amsterdam University Medical Centers, Amsterdam, Netherlands; ^5^Paul-Ehrlich-Institut, Langen, Germany

**Keywords:** allergen standardization, United States, Europe, extracts, major allergen

## Abstract

The topic of standardization in relation to allergen products has been discussed by allergists, regulators, and manufacturers for a long time. In contrast to synthetic medicinal products, the natural origin of allergen products makes the necessary comparability difficult to achieve. This holds true for both aspects of standardization: Batch-to-batch consistency (or product-specific standardization) and comparability among products from different manufacturers (or cross-product comparability). In this review, we focus on how the United States and the European Union have tackled the topic of allergen product standardization in the past, covering the early joint standardization efforts in the 1970s and 1980s as well as the different paths taken by the two players thereafter until today. So far, these two paths have been based on rather classical immunological methods, including the corresponding benefits like simple feasability. New technologies such as mass spectrometry present an opportunity to redefine the field of allergen standardization in the future.

## Introduction

In the European Union (EU), the term standardization commonly relates to product-specific standardization, defined as the pursuit of homogeneity between batches of a single allergen product, or batch-to-batch consistency. “Standardization” in the US encompasses this European definition, but for allergen products also refers to potency, so that allergen extracts from different manufacturers that are derived from the same source (e.g. cat dander) may be compared. This second level of standardization (for sake of distinction sometimes referred to as cross-product comparability), has also been persued in the EU for many years but has not reached the same authoritative character yet as in the US (1). It should be noted that also other countries than the US or EU members also address standardization. For example, Canada requires the use of existing international reference standards when available (2), and South Korea has set up their own allergen standardization initiative which focuses on allergens most relevant for the Korean peninsula [recently briefly summarized by (3)]. While we acknowledge those programs, the United States and Europe will hence be the focus of this review. Regardless of the method, there is general agreement among regulatory authorities and clinicians that the process of standardization improves both efficicacy and safety of allergen immunotherapy.

The vast majority of allergen products are based on extracts prepared from natural allergenic source materials such as mites, plant pollen or animal dander. Because these products are derived from natural sources, they are heterogeneous and variable. Thus, standardization of allergen products has been challenging beginning with the first attempts by Noon, which were based on comparative conjunctival provocation tests in hay fever patients (4, 5). However, in Europe, product-specific standardization has greatly advanced over the last years, due not only to increasing knowledge about individual components that comprise allergenic extracts and technical progress towards measuring those components, but also due to increasing pressure from regulatory authorities and the allergist community (6–10). Consequently, in Europe, more and more allergen product manufacturers nowadays include e.g. the quantification of relevant single allergen molecules in the batch release specifications of their products (6). Nevertheless, most European allergen extracts are standardized for potency according to their capacity to bind IgE in human sera pooled from 10-15 donors.

In Europe, potency of allergen extracts is expressed in arbitrary manufacturer-specific units relative to a so-called in-house reference preparation (IHRP) (11). While the European Medicines Agency (EMA) accepts these manufacturer-specific units for standardization (12), the US Food and Drug Administration (FDA) does not. To facilitate cross-product comparability, the US requires uniform potency-related labelling (13) for each extract for which the Center for Biologics Evaluation and Research (CBER) maintains and distributes reference extracts and serum pools. Thus, while the European system may lead to standardization of more products, it is not possible to compare extracts between manufacturers. Conversely, while the US system allows for comparison of extracts from different manufacturers, it is slow to add to the list of standardized products.

This is one example of differences between the European Union (EU) and the United States with regard to standardization of allergen products. Obviously, the basic foundation for the regulatory environment is different: the uniform situation in the United States under the auspices of the FDA contrasts with the heterogeneity of the current 27 member states in the EU (9). Although there is a common regulatory framework, profound differences exist in its implementation and application among the EU member states and their national competent authorities (9). It should be highlighted though that harmonization efforts are currently pushed forward (14). Currently the European market can be divided into authorized products provided as ready-to-use finished products, and so called named patient products (NPP), manufactured on the basis of an individual prescription and marketed with authorization (15, 16). In the near future, a third category will enter the EU market in the form of allergen extracts standardized with regard to their major allergen content. By contrast, in the US, the market is generally seperated standardized extracts, with defined potency units, and non-standardized extracts, which which are defined concentration of protein or protein nitrogen units.

Apart from regulatory aspects, also the product types and subsequently the clinical practice differ markedly between the US and the EU. In the United States it is common practice that finished products in the form of native allergen extracts are provided to the physicians, who may dilute or mix them according to their patients’ needs before subcutaneous injection. By contrast, while products may contain mixtures of different allergens, physicians do not mix products. In addition, many allergen extracts for injection are adsorbed to aluminum hydroxide or contain other adjuvants, and/or are chemically modified. Also, sublingual immunotherapy is much more popular in the EU than in the USA.

A uniform approach to allergen standardization leading to improved comparability of products from different manufacturers and products authorized by different regulatory authorities has been a goal for decades. However efforts to reach that goal have been impeded by national differences, including differences in market, product types, clinical practice, product-specific standardization, and also regulation. This review will focus on past, current and future efforts in allergen standardization by highlighting and discussing common approaches and differences between the US and EU countries.

## Joint Standardization Efforts

The first official allergen standards in Europe have been prepared in the 1970s in the United Kingdom by the National Institute for Biological Standards and Control (NIBSC) in a newly setup “laboratory for allergens”. They were all designated “non-WHO Reference Material” and cover a large variety of allergen sources, including several mite species (**Table 1**).

**Table 1:** Non-WHO allergen reference materials established by NIBSC in the 1970s.

Allergen Source	Preparation	Status	Availability	Ref.
Cocksfoot Pollen	extract	non-WHO Reference Material	NIBCS 75/506	(17)
Mannan (*C.albicans*)	purified protein	non-WHO Reference Material	NIBSC 76/515	(18)
Mannan (*C.albicans*)	purified protein	non-WHO Reference Material	NIBSC 77/600	(18)
Twelve Grass Pollen	extract	non-WHO Reference Material	NIBSC 77/616	n.a.
*Acarus Siro*	extract	non-WHO Reference Material	NIBSC 77/662	n.a.
*Glycyphagus destructor*	extract	non-WHO Reference Material	NIBSC 77/664	n.a.
*Tyrophagus putrescentiae*	extract	non-WHO Reference Material	NIBSC 78/517	n.a.
*Aspergillus fumigatus*	extract	non-WHO Reference Material	NIBSC 78/575	(17, 19, 20)
*Tyrophagus longior*	extract	non-WHO Reference Material	NIBSC 78/582	n.a.
Honey bee venom	venom	non-WHO Reference Material	NIBSC 78/628	n.a.

n.a., not available.

The respective allergenic materials were either donated to NIBSC from companies (e.g. honey bee venom by Sigma Aldrich) or extracts were prepared directly at NIBSC. The materials have been filtered, filled and freeze-dried. No unitage has been assigned to the materials. They are still available today at NIBSC, though requested at low levels. Unfortunately, some events including inappropriate use of the references in skin prick tests studies lead to the decision of the NIBSC in the 1990s to invest no further laboratory work in allergen references (personal communication).

The first major international effort towards cross-product comparability was a joint activity for establishment of international standards and corresponding methods initiated in 1977 by Alain de Weck. Three years later, this initial group was reorganized, resulting in the formation of the World Health Organization and International Union of Immunological Societies (WHO/IUIS) Allergen Standardization Subcommittee (4). From its foundation until today, the committee members cover all disciplines dealing with allergen standardization: clinicians, scientists, allergen product manufacturers and regulators, from both North America and Europe. The committee’s first major objective was the preparation, characterization and subsequent WHO approval of allergen extract standards. Thanks to exemplary commitment, the group managed to establish eight allergen extract standards within less than a decade (**Table 2**). In parallel to all WHO biological reference materials, each of the allergen extract standards was arbitrarily assigned 100,000 international units (IU). The extracts were stored at -20°C as freeze-dried powders. Five of these allergen extract standards are still available today at the NIBSC (Potters Bar, UK) and can be purchased for £ 126 per ampoule. All extracts had been extensively studied using a broad spectrum of state-of-the-art immunochemical and physicochemical techniques. Although not all participating laboratories performed all methods, and in spite of the use of different versions of the methods, quite consistent results could be obtained. Regarding single allergen molecules, the possibilities for their quantification were still limited. In most cases only relative concentrations could be reported by assigning a concentration of 100% to one of the candidate extracts. However, this was in line with the zeitgeist of the 1980s, where biological standardization was in the focus. Consequently, the central analytical methods in the project were the radioallergosorbent (RAST) inhibition test, measuring overall IgE binding potency, and the crossed immunoelectrophoresis/crossed radioimmunoelectrophoresis (CIE/CRIE) methods, establishing overall protein composition. With very few exceptions, both assays were performed in each laboratory. This was in line with the proposal on standardization of allergenic extracts of the Committee for Allergen Standardization within the Nordic Association of Allergology, first published in 1982 and subsequently revised in 1989 (33). Moreover, as an approach to *in vivo* standardization, skin testing was performed with several reference standard candidates, but patient numbers differed greatly between allergens (ranging from 5 to 46) and two different methods were applied: skin prick test according to the Nordic Guidelines (33) and intradermal testing as developed by Turkeltaub, also referred to as the ID_50_EAL method (15–17). While the Nordic method compares the wheal size with a histamine dose-response curve, the ID_50_EAL method measures the erythema response to determine the ID_50_ value (intradermal dilution for 50 mm sum of erythema; for more details see **Supplemental Data**). Until today, both methods are accepted in the EU to determine biological activity of the IHRP (12), whereas only the ID_50_EAL method is accepted by the FDA (34).

**Table 2:** Allergen extract standards established by the WHO/IUIS Allergen Standardization Committee.

Allergen	Status	Availability	Characterization	Reported allergen content	References
Dog Hair Dander *Canis familiaris*	WHO Int. Standard	NIBSC 84/685	15 laboratories in 9 countries protein content RAST inhibition CIE/CRIE/RIE IEF SDS-PAGE leukocyte histamine release HCCT RMDT skin testing ()?*	100 µg Can f 1/ampoule	(21, 22)
Short Ragweed pollen *Ambrosia artemisiifolia*	WHO Int. Standard	NIBSC 84/581	12 laboratories in 5 countries protein content RAST inhibition CIE/CRIE TLIEF leukocyte histamine release HPLC intradermal skin testing (n=5)	26-40 µg Amb a 1/ampoule (85-133 µg Amb a 1/ml)	(23)
Birch pollen *Betula verrucosa*	WHO Int. Standard	NIBSC 84/522	20 laboratories in 11 countries protein content RAST inhibition CIE/CRIE/RIE IEF SDS-PAGE leukocyte histamine release skin prick testing (n=20)		(24)
Timothy grass pollen *Phleum pratense*	WHO Int. Standard	NIBSC 82/520	14 laboratories in 10 countries protein content RAST inhibition CIE/CRIE/RIE IEF SDS-PAGE HPLC ELISA-inhibition complement inactivation leukocyte histamine release		(25)
House Dust Mite *Der. pteronyssinus*	WHO Int. Standard	NIBSC 82/518	19 laboratories in 11 countries RAST inhibition CIE/CRIE/RIE/RIA/SRID IEF intradermal skin testing (n=3) skin prick testing (n=43) leukocyte histamine release ELISA inhibition direct RAST RMDT HCCT	12.5 µg Der p 1/ampoule 0.4 µg Der p 2/ampoule	(26–29)
Bermuda grass *Cynodon dactylon*	International Standard	no longer available	11 laboratories protein content RAST inhibition CIE/CRIE IEF SDS-PAGE leukocyte histamine release		(30)
Alternaria *Alternaria alternata*	International Standard	no longer available	30 laboratories protein content RAST inhibition TLIEF HPLC SDS-PAGE/Western Blot CIE/CRIE/RIE/SRID leukocyte histamine release direct RAST skin testing (n=9)		(31)
Rye grass *Lolium perenne*	International Standard	no longer available	6 laboratories RAST inhibition protein content CIE/CRIE SDS-PAGE/Western Blot IEF skin testing (?)+ ELISA inhibition		(32)

*stated on official NIBSC leaflet, but not mentioned in publications.

+statement in (26) that skin testing had been performed, but respective data is not provided.

CIE, crossed immunoelectrophoresis; CRIE, crossed radioimmunoelectrophoresis; HCCT, human complement consumption test; HPLC, high performance liquid chromatography; RIA, radioimmunoassay; RIE, rocket immunoelectrophoresis; RMDT, rat mastocyte degranulation test; SRID, single radial immunodiffusion; TLIEF, thin layer isoelectricfocusing.

Despite their intensive characterization and the broad spectrum of researchers, regulators and allergen product manufacturers involved, the standard extracts listed in **Table 2** never became broadly accepted or used. Several factors contributed to this unfortunate situation. Firstly, many allergen product manufacturers did not accept the importance of standardization and especially the central role of major allergen molecules at the time (4). Admittedly, manufacturers were probably also reluctant to make use of the reference standards as long as this remained non-mandatory. One has to remember that allergen products had only just become part of European pharmaceutical legislation in 1989 and a concurrent regulation imposing the use of the new standards was seen as unfeasible.

Secondly, the FDA did not fully agree with the WHO/IUIS standardization approach at the time, because skin testing was regarded to be the only acceptable basis for standardization. The matter has been a point of discussion in the WHO/IUIS Allergen Standardization Subcommittee for many years after, but there was no turning back. Subsequently standardization efforts in Europe and the USA drifted apart.

## Allergen Standardization in the USA

### Regulatory Background in the USA

Allergen extracts and other biologics were first regulated by the Hygienic Laboratory of the Public Health and Marine Hospital Service. In 1930, the Hygienic Laboratory was renamed the National Institute (singular) of Health (NIH). The NIH continued to regulate biologics (beginning in 1955, through its Division of Biologics Standards) for over forty years. In 1972, regulatory authority over biologics was transferred to the Bureau of Biologics at the Food and Drug Administration (FDA). In 1982, the FDA merged the Bureau of Biologics and the Bureau of Drugs into a single National Center for Drugs and Biologics; five years later, the entities that regulated drugs and biologics were once again separated, and the Center for Biologics Evaluation and Research (CBER) assumed responsibility for regulation of allergenic extracts (35, 36).

CBER’s authority to regulate allergen extracts is derived from two federal laws, the Food Drug and Cosmetic Act of 1938 and the Public Health Service Act of 1944, as amended. The specific regulations that govern CBER’s regulation of allergens appear in part 680 of Title 21 of the Code of Federal Regulations (21 CFR 680), although other parts of 21 CFR also apply to allergen regulation. Over the past several decades, two features of CBER’s regulatory program have had a significant impact on allergen manufacturers and enhanced the safety of allergen extracts marketed to the American public. The first feature is the enforcement in the 1960’s of current good manufacturing practice (cGMP) standards (21 CFR 210, 211, and 600-680) on the manufacture of allergen products. cGMPs include requirements regarding organization and personnel, buildings and facilities, equipment, control of components and drug product containers and closures, production and process controls, holding and distribution, quality control, laboratory controls and records and reports.

The second feature of significant impact in 21 CFR 680 is allergen standardization.

### CBER Reference Materials

As outlined above, the purpose of allergen standardization is to characterize the potency of allergen extracts and minimize the variation between lots of allergen extracts (product-specific standardization), or even among different manufacturers (cross-product comparability). Since the 1980’s, 19 allergen extracts have been standardized (**Table 3**). While the set of standardized allergens is a small fraction of the total number of allergen extracts sold in the US, it constitutes a substantial fraction of environmental allergens that are used in allergen immunotherapy. The level of quality control for the 19 standardized allergen extracts is the exception rather than the rule. Without *in vitro* potency tests that correlate with *in vivo* clinical responses, the consistency of non-standardized extracts cannot be ensured.

**Table 3:** Standardized allergen extracts currently licensed in the US.

Allergen extract	Lot release tests	Labeled Unitage	Year standardized
**Dust mite *(Dermatophagoides farinae)***	cELISA Protein*	AU/mL	1987-1989
Dust mite *(Dermatophagoides pteronyssinus)*			
Cat pelt (*Felis domesticus)*	Fel d 1 (RID) IEF Protein*	BAU/mL^†^	1992
Cat hair (*Felis domesticus)*			
Bermuda grass *(Cynodon dactylon)*	cELISA IEF Protein*	BAU/ml	1997-1998
Red top grass (*Agrostis alba)*			
June (Kentucky blue) grass (*Poa pratensis)*			
Perennial ryegrass (*Lolium perenne)*			
Orchard grass (*Dactylis glomerata)*			
Timothy grass *(Phleum pratense)*			
Meadow fescue grass *(Festuca elatior)*			
Sweet vernal grass (*Anthoxanthum odoratum)*			
Short ragweed (*Ambrosia artemisiifolia)*	Amb a 1 (RID)	Amb a 1 units	1981
Yellow hornet (*Vespa* spp)	Hyaluronidase & phospholipase activity	µg protein	1991-1995
Wasp (*Polistes* spp)			
Honey Bee *(Apis mellifera)*			
White faced hornet *(Vespa* spp)			
Yellow jacket (*Vespula* spp.*)*			
Mixed vespid (*Vespa + Vespula* spp)			

cELISA, competitive ELISA; IEF, isoelectric focusing; BAU, bioequivalent allergy unit; AU, allergy unit (equivalent to BAU).

*Test for informational purposes only. IEF, isoelectric focusing;

^†^For Cat Pelt and Hair extracts: 5-9.9 Fel d 1 U/mL = 5000 BAU/mL; 10-19.9 Fel d 1 U/mL = 10,000 BAU/mL.

21 CFR 680.3(e) requires that the potency of each lot of Allergenic Product be determined, and that potency test methods must measure the allergenic activity of the product. This regulation establishes a US standard of potency for each standardized product and mandates that manufacturers must state the potency on the label of each vial. 21 CFR 680.3(e) also specifies that once a potency test exists for a specific allergenic product and CBER has notified manufacturers that the test exists, manufacturers must determine the potency of each lot of the product prior to release. An important distinction between the US and Europe is that rather than the manufacturers, it is the regulatory authority, CBER, who specifies whether potency tests will be done, which test defines potency, and the unitage by which potency is defined. To facilitate compliance with the standardization requirements, CBER maintains a reference reagent program to provide reference reagents to manufacturers for potency testing in which stocks are maintained and reagents are replaced when stocks are depleted. Rather than use CBER’s reference reagents, manufacturers may seek approval to use an alternative test method that provides an equally reliable measure of product potency and meet regulatory requirements. Regardless of the test, however, manufacturers must use the unitage of potency that CBER assigned to the product.

### Assigning Potency

The choice of the best potency test depends on the allergen extract to be standardized. While Europeans strive towards using major allergens to define potency (37), CBER assigns one major allergen as the potency unit only to short ragweed pollen and cat hair extracts, and two allergens each to two additional extracts, cat pelt (Fel d 1 and albumin) and Hymenoptera venom (hyaluronidase and phospholipase A2 for Hymenoptera venoms). Although Amb a 1 and Fel d 1 are measured by radial immunodiffusion assay, CBER will replace this method for the enzyme-linked immunosorbent assay (ELISA). Concentrations of hyaluronidase and phospholipase A2 are measured by measuring their enzymatic activity.

When data do not sufficiently support assigning potency to a dominant allergen, as is the case for house dust mite (HDM) and grass pollen extracts, CBER uses a measure of “overall allergenicity.” To assign units of overall allergenicity, CBER developed a method of intradermal testing of highly allergic individuals with serial dilutions of extract that uses the size of erythema in response to intradermal injection. Intradermal injection was chosen over prick/puncture testing to achieve greater dosing accuracy; erythema size was chosen over wheal size to achieve greater accuracy in reaction measurements (38). This method is called “IntraDermal dilution for 50 mm sum of Erythema determines the bioequivalent ALlergy units” (ID_50_EAL) and can be used to compare the allergenicity of extracts regardless of manufacturer. For grass pollen extracts, the unitage is “bioequivalent allergy unit” (BAU); For HDM, the unitage “allergy unit” (AU) was originally assigned and has been retained. Subsequent comparisons of extracts from the same source material are made by a variant analysis called the parallel line bioassay. ID_50_EAL and parallel line methods are described in detail in **Supplemental Data**.

Although skin testing was essential to development of the allergen standardization program, it is not feasible for routine lot testing. For that purpose, surrogate *in vitro* potency assays that accurately predict the *in vivo* activity of extracts have been developed (39). For grass pollen and HDM extracts, the surrogate test is competition ELISA that measures inhibition by the newly manufactured test extract to inhibit binding of IgE from pooled allergic sera to a reference allergen (grass pollen, HDM) (40).

As described above, potency units for short ragweed pollen extracts were originally assigned based on their Amb a 1 content as units of Amb a 1 (also called Antigen E), subsequent data indicated that 1 unit of Amb a 1 is equivalent to 1 μg of Amb a 1. While ID50EAL testing showed that 350 Amb a 1 units/mL is equivalent to 100,000 BAU/mL, the original unitage of Amb a 1 units has been retained. Cat extracts, however, were originally standardized based on their Fel d 1 content as AU/mL. Subsequent ID50EAL testing resulted in the assignment of 10,000 BAU/mL unitage to cat extracts, which contained 10-19.9 Fel d 1 U/mL (41). In addition, since 20% of individuals allergic to cat were found to have antibody to non-Fel d 1 proteins (42), showing that the extract contains albumin (Fel d 2) by isoelectric focusing (IEF) was added as a requirement for cat pelt extracts.

### Future Activities

Allergen standardization has led to a core group of highly used allergen extracts that are more consistent than their non-standardized predecessors. As we move forward towards standardizing more of the currently licensed non-standardized extracts, it has become apparent that standardizing to an immunodominant allergen is restricted by the limited number of allergen sources for which there is uniform consensus of an immunodominant allergen, and standardizing according to overall potency fails to account for the explosive body of literature in which many allergenic proteins have been defined and categorized.

To overcome these limitations, CBER researchers are developing novel approaches towards determining allergen extract potency with the goal of assessing overall potency of complex allergen mixtures as the integral of multiple discrete allergen assays. A promising novel method is tandem mass spectrometry (MS), which precisely measures quantities of signature peptides for each allergen (43, 44). Such detailed characterization of complex extracts invites the possibility of matching the precise characterization of extract components with the emerging use of component resolved diagnostics to personalize allergen immunotherapy and further enhance its safety and efficacy.

## Allergen Standardization in the EU – Activities and Current Status

### Regulatory Background in Europe

Prior to 1989, regulation of allergen products in Europe solely depended on the respective national legislation in every member state. Some products for allergen-specific immunotherapy aquired national marketing authorizations (MA) or registration based on the respective national licensing procedures, but the vast majority of products were NPPs (45, 46) Harmonized legisalation with regard to allergen products started in the EU in 1989 based upon Directive 89/342, which extended the scope of Directives 65/65/EEC and 75/319/EEC and thereby demanding registration of allergen products as medicinal products (47, 48). This entails the requirement of compliance with Good Manufacturing Practice (GMP) as well as submission of clinical data to demonstrate safety and efficacy. To help both manufacturers and NCAs in implementing the necessary concepts, the first Note for Guidance on Allergen Products was issued in 1992 by the Committee for Proprietary Medicinal Products (CPMP) and revised in 1996. The resulting document (CPMP/BWP/243/96) was in line with the first Monograph on Allergen Products published in 1997 in the Ph. Eur. In 2001, the Directive 2001/83/EC came into force, representing a key document of European legislation, also in relation to allergen products. It defines them as “any medicinal product which is intended to identify or induce a specific aquired alteration in the immunological response to an allergizing agent” (49). Consequently, allergen products both for therapeutic and diagnostic use require an MA in the EU, as long as the products are industrially prepared or manufacturing involves an industrial process step. However, the implentation of the Directive 2001/83/EC is still heterogeneous among member states to this day and the regulatory environment in the EU is complex, with each member state having its own independent national competent authority. Despite the availability of harmonized European procedures, most allergen products authorized until today have undergone a national MA procedure. For a complete overview on the current regulatory system in the EU, including details on the different types of MA procedures, please see Bonertz et al. (9).

Specific guidance documents for allergen product manufacturers have been laid down in the European Pharmacopoeia in the Monograph on Allergen Products (50) and in the Note for Guidance on Allergen Products (51), which was replaced in 2008 by the Guideline on Allergen Products: Production and Quality Issues (12). Importantly, both the Monograph and the Guideline state that the concentration of relevant individual allergens should be determined, if possible, using certified reference standards or biological reference preparations and assays validated in international standardization programs. However, the way to establish the necessary materials on a European level has been and still is laborious.

### Transition From Extracts to Molecules

It was in the 60s of last century that the first major allergens were identified in allergenic pollen extracts, starting with Antigen E (now called Amb a 1) in ragweed (52), shortly followed by Rye I, II and III (now called (Lol p 1, 2 and 3) in ryegrass (53). In the mid-70s, the first major allergens of bee and wasp venom were characterized (54), and in the 80s the two most important house dust mite allergens Der p 1 (55) and Der p 2 (56), and the major allergen from cat (57, 58) were purified and characterized. In 1988, the first two major allergens, Der p 1 from house dust mite (59) and Bet v 1 from birch pollen (60), were cloned. Ever since that time, many hundreds of major and minor allergens from a broad spectrum of allergen sources have been identified, purified, cloned and/or expressed (61, 62). In parallel, specific antibody reagents, both monoclonal and polyclonal, were developed allowing quantification of major allergens by immunoassays such as sandwich ELISAs (63). Towards the turn of the century, for the most important pollen and indoor allergen sources, major allergens had been identified and often multiple specific assays for their quantification were available. The dominant role of major allergens in allergic disease has stressed their importance as active pharmaceutical ingredients of immunotherapy products that need to be present at optimally effective dosages. Slowly, this has moved the field to more and more extend their standardization efforts towards quantification of major allergens, besides overall IgE potency determinations and SDS-PAGE profiles. At the turn of the century, measurement of major allergens in most important allergen sources was within reach. Most allergen manufacturers however used, and until now still use, their own unique in-house units, linked to some form of IgE potency measurement. IgE potency measurements are typically carried out by competitive immunoassays such as ELISA inhibition or ImmunoCAP inhibition. In these assays, serum pools composed of sera from patients allergic to the source are used. Because of their finite nature, composition changes with time, and composition differs between allergen manufacturers. The composition of serum pools will influence the sensitivity by which different major allergens are picked up and consequently determine the overall potency.

### The CREATE Project

**Figure 1:**
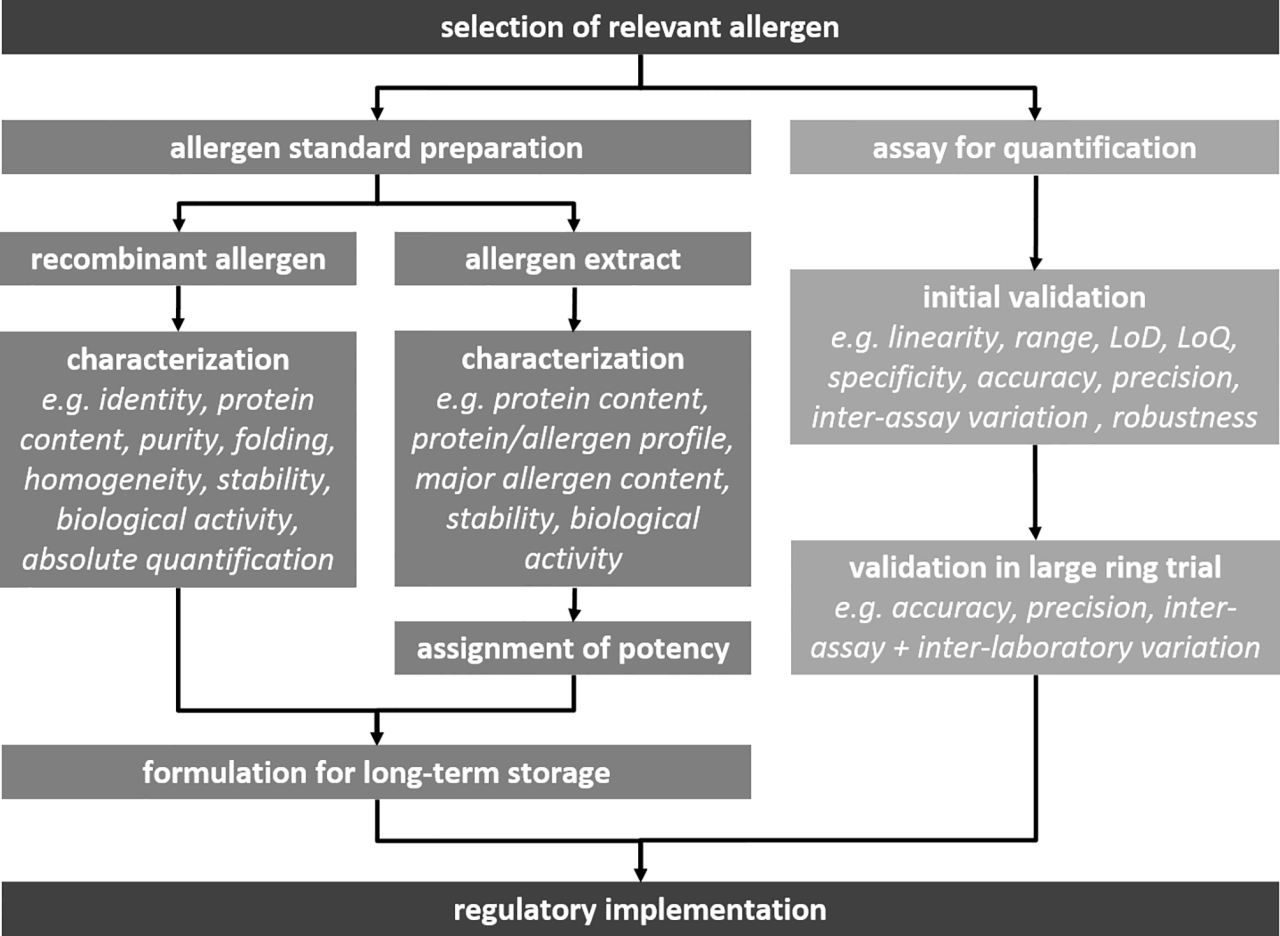
Steps in the development of allergen standard preparations and methods in the EU.

The realization that sufficient (efficacy) and consistent (safety) presence of major allergens is decisive for the quality of immunotherapy products, stressed the need to allow direct comparison between competitor products with respect to major allergen content (64). In 2001, a consortium of allergen manufacturers, academic research institutes, clinical researchers and regulators joined forces in an EU-funded project, the CREATE project. Tackling the first steps necessary in performing international allergen standardization (**Figure 1**), the CREATE consortium evaluated recombinant allergens from house dust mites, grass pollen, birch pollen and olive pollen for their appliability to serve as biological standards for major allergen quantification. For each of the eight selected allergens (Der p 1, Der p 2, Der f 1, Der f 2, Phl p 1, Phl p 5, Bet v 1 and Ole e 1), multiple sandwich ELISAs were compared to allow future selection of assays that could potentially serve as reference assays linked to recombinant allergen standards. Clinical centers participating in the consortium collected serum samples from patients with confirmed allergy to the four allergen sources. These serum samples were used to investigate whether the recombinant allergens were immunologically approriate mimics of their natural counterparts. Each of the eight allergen molecules, natural and recombinant, was subjected to a detailed physico-chemical charatcerization protocol, to establish identity, purity, correct folding and aggregation status (65). Some of the recombinant allergens, such as the mite group 1 allergens and the grass pollen group 1 allergen, proved to be poor mimics of their natural counterparts, both physico-chemically and immunologically (37, 66). Sandwich ELISA evaluations revealed that some did not pick up all natural isoforms, and in other cases standard curves did not run parallel to dilution series of allergen extracts. In the end, recombinant allergen standards and some associated ELISAs performed best in case of Bet v 1 and Phl p 5. They were identified as good candidates for further development towards estblishment as official biological standards.

### BSP090

**Figure 2:**
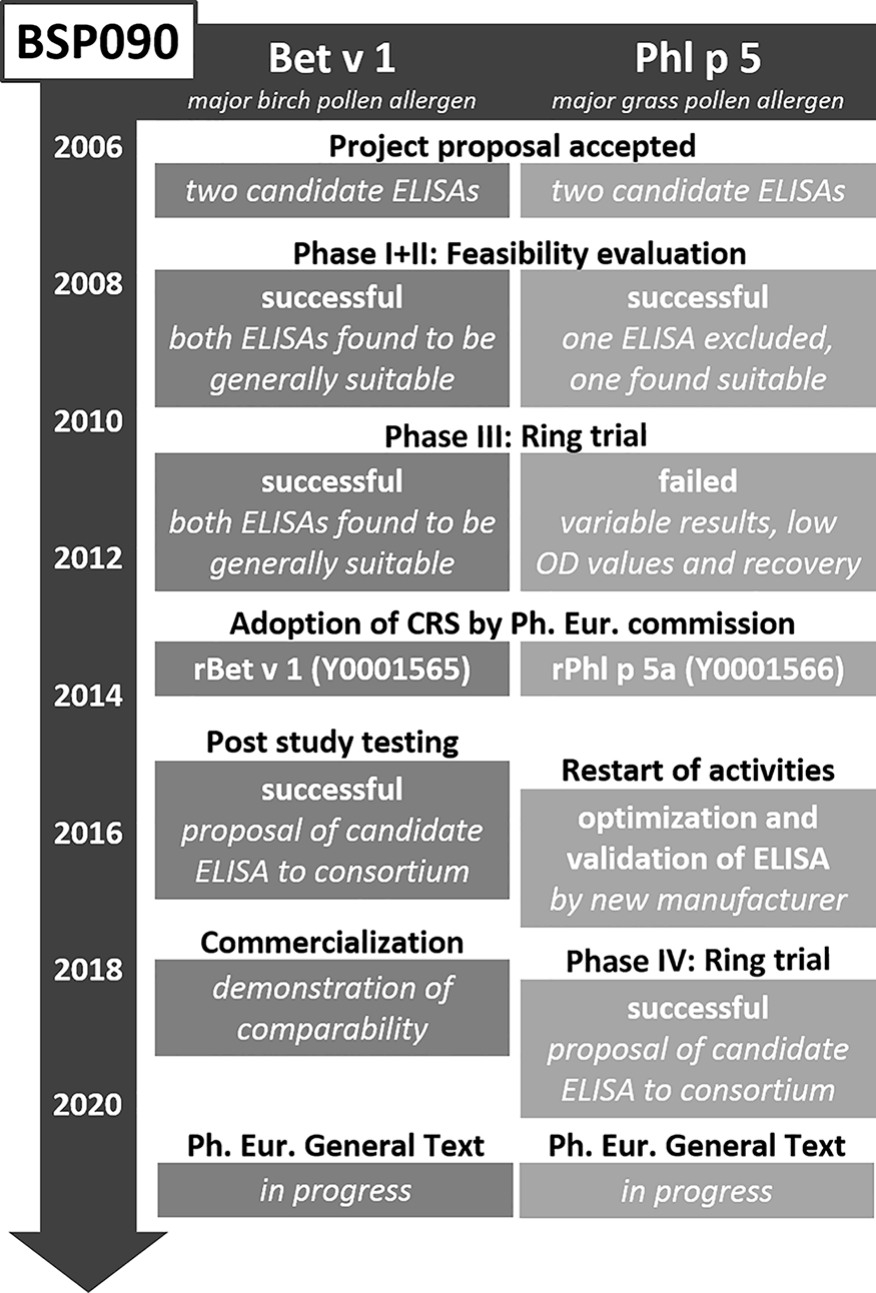
Overview of the BSP090 project.

After finalization of the CREATE project in 2005, a follow-up project was initiated. BSP090 was part of the Biological Standardisation Programme of the European Directorate for the Quality of Medicines & Health Care (EDQM). It focused on two major allergens, namely Bet v 1 from birch pollen and Phl p 5 from timothy grass pollen, which had been identified in CREATE as most promising candidates. The decision to limit the project to only two allergens turned out to be correct as the goal of establishing two Ph. Eur. chemical reference substances (CRS) in conjunction with two Ph. Eur. standard methods for quantification has still not been fully reached (**Figure 2**). The two recombinant proteins rBet v 1.0101 and rPhl p 5.0101 were produced under GMP conditions and intensively analyzed using an array of physicochemical and immunological methods to obtain information on identity, quantity, homogeneity, fold stability in solution, and biological activity. In addition, formulated versions of the allergens for long-term storage were assessed for thermal denaturation, aggregation state, and biological activity (67, 68). In 2012 these two CRS preparations became adopted by the Ph. Eur. Commission and available at the EDQM for purchase (EDQM catalogue numbers Y0001565 and Y0001566). However, their use has been so far rather limited as it has not become mandatory yet. Until now, the Monograph on Allergen Products only states that allergen-specific reference standards may be used, when available (50). This will change upon adoption of the corresponding standard methods, but the way to this goal proved unexpectedly time-consuming. After successful completion of two feasibility study phases, two Bet v 1-specific and one Phl p 5-specific ELISA system were included in an international ring trial in 2010 (69, 70). Model samples containing the respective allergen extract spiked with recombinant protein, were assayed in 13 laboratories in the USA and Europe. Results for both Bet v 1-specific ELISAs were promising. Based on these findings and a post-study testing with a large set of birch pollen allergen products, one of the ELISAs was selected to become standard method. Unfortunately, the results for the Phl p 5-specific ELISA were not satisfactory and it was not until 2018 that a second international ring trial could be initiated with an updated version of the ELISA. The data collected in 13 participating laboratories was considered appropriate to recommend the ELISA as second standard method (71). Implementation of the Bet v 1 and Phl p 5 ELISA protocols as general chapters in the Ph. Eur. is currently in progress.

### Current and Future Activities

Once the two standard methods have been implemented in the Ph. Eur. and the Monograph on Allergen Products has been revised, the use of both CRS and standard method will become mandatory for allergen product manufacturers in the EU. This will, for the first time, enable cross-product comparability of birch pollen and timothy grass pollen allergen products based on major allergen content. As it can thus be expected that the demand for the two allergen CRS will increase in the years to come, a new project has been initiated as part of the Biological Standardisation Programme of the EDQM called BSP163. In the course of this project, new batches of rBet v 1 and rPhl p 5a will be analysed at the EDQM, the University of Salzburg and the Paul-Ehrlich-Institut to prepare and qualify second CRS batches for both allergens.

Furthermore, after completing BSP090, the allergen standardization sub-committee has decided to initiate a new project to proceed in establishing further allergen standards and corresponding quantification methods. The first phase of the project will be a public call for both commercial and non-commercial allergen specific ELISA methods available as well as candidate allergen standards, focussing on several potential candidate allergens including group 1 and group 2 allergens from HDM, Ole e 1 from olive pollen and Ara h 1/Ara h 2 from peanut. The latter will to our knowledge represent the first attempt of international standardization of a food allergen. The starting situation is basically promising: Food allergy is generally of great and constantly increasing importance (72, 73) and a large number of relevant food allergens are known for many food sources, as detailed in the database of the WHO/IUIS Allergen Nomenclature Subcommittee (allergen.org). In addition, the first immunotherapy product for treatment of peanut allergy has gained marketing approval in the USA and EU, and further products are under review and in development. Also, the respective pharmaceutical companies have established methods for quantification of single relevant allergens. Although standardization relating to batch-to-batch consistency is thus ensured for these products upon marketing authorization approval (74), establishment of international standards and standard methods aiming at comparability between different products is an important goal.

## Potential of Alternative Methods in Allergen Product Standardization

### Mass Spectrometry for Analysis of Allergen Preparations

As described above, the high heterogeneity in protein and allergen content of diverse allergen products is abundantly documented in the literature posing a challenge for the standardization of such products and their clinical use. Major problems are caused by insufficient amounts or absence of allergens, but may also be caused by unusually high amounts of certain allergens as demonstrated for LTPs in olive pollen AIT products (75). As outlined, traditionally, quantitative *in vivo* assays (e.g. intradermal skin testing) (38) or *in vitro* immunoassays (e.g. ELISA) using monoclonal, or polyclonal antibodies, or patients’ IgE have been used for detection and quantification of allergens. However, using patient IgE-based potency assays only provide a measure of overall potency, without information on single allergenic components. In contrast, allergen-specific immunoassays are ususally limited to one allergen at a time and thus allergen sources with several major allergens are difficult to standardize. Although allergen-specific multiplex ELISAs as e.g. available for indoor allergen quantification (76) might circumvent this limitation for the user, standardization of multiplex ELISAs is challenging. Thus, alternative analytical tools providing accurate, sensitive, and fast analyses are increasingly demanded for standardization and regulation of commercial allergen products.

Several physicochemical methods like fluorescence spectroscopy, far-UV circular dichroism, Fourier-transform infrared spectroscopy (FTIR) and online-high performance size exclusion chromatography (HPSEC) light scattering have been shown to provide insight into questions not addressed by immunoassays, like information on protein structures or molecular weight distributions in allergen extracts (77–79). In comparison, mass spectrometry (MS) has the potential to replace standard immunoassays due to its high accuracy not only for detection but also for quantification of allergenic proteins in complex samples (80, 81). MS systems are normally defined according to the different types of their three basic components, *i.e*. ion source, mass analyzer, and detector. The most frequently used types of ion source are matrix-assisted laser desorption/ionization (MALDI) and electrospray ionization (ESI), whereas time-of-flight (TOF), quadrupole (q), ion trap (IT), and Orbitrap are commonly used as mass analyzers in proteomics. The combination of different ion sources and mass analyzers gives rise to hybrid MS devices, such as ESI-qTOF, ESI-IT, MALDI-TOF, or ESI-qOrbritrap. In addition, MS devices can be combined with high performance liquid chromatography (LC-MS), which improves resolution and facilitates identification and quantification of peptides. Initially, MS techniques providing highly accurate mass determinations (e.g. MALDI, qTOF, qIT) have been used to study the isoform composition of major allergens in natural sources (82–84), to identify novel allergens (85–87), or to assess structural integrity of recombinant allergen preparations such as Bet v 1 (68) and Phl p 5 (67). A more recent MS approach for clinical applications and allergen analysis is based on the use multiple reaction monitoring (MRM) systems. In this respect, MRM–based targeted proteomics using internal standards seem to be a particularly suitable option for allergen standardization due to its wide linear dynamic range, high intra- and inter-assay precision, and broad potential of multiplexed analysis (88). In fact, a comparative analysis between several commercially available ELISA tests and the MRM-based assay showed that ELISA kits underperformed in the quantification of multiple allergens in processed bakery products (89). Depending on the respective system, major limitations of non-MS-based methods can be cross-reactivity, narrow quantification range and/or poor reproducibility. Thus, MS-based MRM has emerged as a powerful approach for the rapid establishment of quantitative assays with high specificity, precision, and reproducibility (90, 91). One disadvantage of MRM is the lower accuracy of mass determination when compared with other MS systems (80). However, the great advantage is the precise quantification of target proteins with highly variable concentrations, such as those of allergenic proteins in commercial products. MRM analyses are mainly performed on instruments combining high performance liquid chromatography (*e.g.* nano-LC, HPLC, UUPLC) with triple quadrupole MS instruments. Four major steps are carried out during MRM experiments: (i) precursor mass selection at the first quadrupole analyser, (ii) fragmentation of selected precursor mass, (iii) scanning of fragment ions of interest, and (iv) quantification of fragment ion. For reproducible and accurate quantification of allergens by MRM MS, the signal intensities from the precursor ion of the endogenous peptide are compared to the precursor ion of the synthetic stable-isotope (^13^C or ^15^N)-labeled peptide standard of known abundance (reviewed in (88, 92). For the selection of peptide standards, untargeted analysis with high-resolution MS instruments is carried out to identify peptides fulfilling a number a criteria, including sequence-based features (e.g. not prone to missed proteolytic cleavages and modifications; precursor ion’s charge, preferably doubly charged), and specificity, including the issue of multiple protein isoforms (93).

The MRM approach has been successfully used for identification and quantification several allergens in extracts prepared from timothy grass pollen (94), cockroach (43, 93), house dust mites (44), mouse urine (95), and to quantify milk, soy, peanut, fish, and egg allergens in several food products (96–101) reviewed in (91, 92). The broad applicability of MS-based MRM was further demonstrated by Mindaye et al. in proof-of-concept studies (43, 93), demonstrating the accurate quantification of German cockroach allergens in complex extracts. As a first step, the authors used an *in silico* prediction together with high-resolution MS for peptide mapping and for the selection of the best representative peptides to serve as standards in the quantification analysis. In total, 26 peptides covering all recognized (n=11) German cockroach allergens/isoallergens were identified and heavy-isotope labeled analogous synthesized for the MRM method development and optimization.

Despite these encouraging findings, very limited information exists for systematic allergen profiling (e.g. biological and clinical relevance of allergens in various sources) and panels of signature peptides are still lacking for absolute quantification of allergens in complex preparations. Thus, for the full implementation of targeted proteomics approaches in allergen standardization further research is needed to establish databases of defined signature peptides of different allergens and allergen sources. Even more research and dedication will be necessary to enable cross-product comparability based on MS in native extracts. Although the Ph. Eur. contains a general instruction on MS (102), no European standard methods have so far been based on MS. The corresponding challenges in relation to allergen product standardization are various. First of all, the establishment of an international standard method will be challenging due to the many different types of MS technologies available, in combination with several different brands per type. In addition, it is likely that different product matrices, polymerization agents and different product processing steps will present a problem in validation of a future MS standard method. Furthermore, it will be necessary to establish allergen-specific reference peptides in combination with a common database to enable comparable results. Moreover, differences between commercial software for MS data analysis may further impact results of database searches. Last but not least, compared to immunoassays, MS provides extensive in-depth information on allergen extracts, *e.g*. in relation to different allergen isoforms. While a standard method based on an immunoassay provides only one result, e.g. on Bet v 1 content in a birch pollen extract, depending on the isoform-specificity of the respective antibodies, MS technologies are able to detect numerous Bet v 1 isoforms in the same extract sample (103). Thus, data collection and interpretation guidelines will be needed to allow cross-product comparability of allergen products based on MS.

### Methods for Analysis of Modified Allergen Preparations

Another issue in allergen product standardization is that in chemically modified and/or adsorbed allergen extracts epitopes may not be readily available for antibody binding causing a decrease in sensitivity of immunoassays, if these have not been specifically tailored to the respective modified allergen. Consequently, most analyitcal methods described so far in this review in allergen product standardization are limited to the analysis of native allergen extracts. At least for the European market this commonly prevents the analysis of the finished product. However, the Guideline on Allergen Products requests the control of consistent quality also after modification, including the demonstration of potency and presence of relevant allergens (12). Both research groups as well as allergen product manufacturers have developed a number of such methods (6). Notably, these methods are so far either used in a scientific context or for in-depth analysis of commercial products by allergen product manufacturers, *e.g*. to control batch-to-batch consistency. Their potential suitability for future cross-product comparability has hardly been considered. **Table 4** provides an overview of methods for standardization of chemically modified and/or absorbed allergen products, including examples of their use as well as an assessment of their potential suitability for cross-product comparability. As becomes apparent from **Table 4**, there are currently no examples of absolute quantification of single allergens in allergoids. Published examples of product-specific standardization in allergoids are either based on IgGs raised against an allergoid in animal models determining overall IgG potency or on MS for confirmation of presence of singe relevant allergens. Given the challenges encountered for native allergen extracts, the goal of cross-product comparability in allergoids is currently out of reach.

**Table 4:** Analytical methods for standardization of chemically modified and/or absorbed allergen products.

Method		Examples	Ref.	Cross-product comparabiltiy?
**IgG inhibition ELISA**		potency determination adsorbed HDM allergoid	(104)	- necessity of allergen-specific allergoid reference standard and allergoid-specific reference method
		potency determination grass pollen, birch and HDM allergoids	(105)	- depending on specificity of antibodies
		potency determination cat dander allergoid (IgG in patient sera pool)	(106)	- potentially challenging due to different product matrices, polymerization agents and additional product processing steps
**MS**	**LC-MS/MS**	HDM allergoid, confirmation of presence of major allergens	(107)	- potentially challenging due to different product matrices, polymerization agents and additional product processing steps
	**LC-MS/MS**	depigmented and aluminium hydroxide-adsorbed birch pollen extract, confirmation of presence of allergens	(79, 108)	- challenging due to different types of MS + brands of machines - necessity of allergen-specific reference peptides
	MS*	(adsorbed) HDM allergoid, identification of relevant allergens	(78)	- necessity of common database
**IgG induction rabbits**		induction of specific IgG to Bet v 1 and Bet v 2 after immunization with depigmented and aluminium hydroxide-adsorbed birch pollen extract	(108)	- in conflict with the principles of 3R (109)
				- limited reproducibility

*type of MS unclear from information provided in publication.

## Discussion/Conclusion

Although the joint standardization efforts in the early 1980s had the goal and potential to build a common basis for allergen standardization in the US and Europe, this has unfortunately not been achieved. Instead, the ways towards cross-product comparability have been drifting apart for decades, though with differing success (**Table 5**).

**Table 5:** Comparison of reference standards and methods in the United States and Europe.

	United States	Europe	
**number of allergen standards**	19 standard extracts (see **Table 3**)	2 rec. allergens	5 standard extracts (see **Table 2**)
**unitage of standards**	potency (depending on allergen, see **Table 3**)	µg single allergen molecule	international units
**responsible authority**	FDA/CBER	EDQM/NCAs	
**main regulatory document**	21 CFR 680.3(e)	Ph. Eur. (Monograph on Allergen Products)	
**provision of reference method materials**	CBER	commercial sources	
**provision of reference standards**	CBER	EDQM	
**mandatory use**	yes	(yes)*	
**option to use alternative method**	yes	yes	

*after adoption of respective general texts in Ph. Eur.

Based on a great effort undertaken in the 1980s and 1990s, a panel of 19 standardized allergen extracts has been available in the US for more than 25 years. Although these do not cover all allergen sources relevant for the US population, the reference materials have clearly helped to increase consistency of allergen extracts on the US market (110). In contrast, allergen standardization took a while to get back to its feet in Europe after it became clear that the effort of establishing eight standard extracts had been more or less in vain. In addition, the re-start of activities, firstly in CREATE and subsequently in the project BSP090, proved to be unexpectedly challenging. After 20 years, only two recombinant major allergens and the corresponding reference ELISA methods have been validated and are on their way to be included in the Ph. Eur. This will for the first time allow for a direct comparison of birch pollen and Timothy grass pollen allergen products with regard to their major allergen content in the EU. Although many lessons have been learned on the way, the establishment and validation of ELISAs for selected major allergens is laborious. While in the 1980s there was a great willingness to support projects aiming at cross-product comparability, the necessary resources are far more difficult to aquire nowadays. Nevertheless a new project will soon be initiated to identify suitable candidate references and candidate assays for further important allergens.

In view of the different paths taken by the US and Europe, it seems rather unlikely that using current technology, there will be a harmonized approach for allergen standardization. However, ongoing active research at CBER and at the Paul-Ehrlich-Institut aimed towards using MS to standardize allergen extracts offers hope towards harmonization between the US and Europe (44), although a number of questions must be addressed before allergen product standardization *via* MS can be implemented.

## Author Contributions

JZ: drafting of chapters 1,2, 4.4, 4.5, 5.2 and 6. JB: drafting of chapters 3 and **Supplemental Material**. FF: drafting of chapter 5.1. RvR: drafting of chapters 4.1, 4.2 and 4.3. RLR: drafting of chapters 3 and **Supplemental Material**. SV: drafting of chapters 1,2, 4.4, 4.5, 5.2 and 6. All authors contributed to the article and approved the submitted version.

## Funding

No funding was received for the preparation of this review article. The standardization project CREATE, BSP090 and BSP163 described in this review article were funded in part by the 5th Framework Programme of the European Union (Contract No. G6RD-CT-2001-00582), the BSP090 grant (EDQM) and the BSP163 grant (EDQM).

## Author Disclaimer

RLR and JB: This article is an informal communication that represents the authors’ judgment. These comments do not bind or obligate FDA. SV and JZ: This article is an informal communication that represents the authors’ judgment. These comments do not bind or obligate the Paul-Ehrlich-Institut.

## Conflict of Interest

The author FF declares personal fees from HAL Allergy, from the Swiss Institute of Allergy and Asthma Research (SIAF), and from AllergenOnline, all outside the submitted work. The author RvR declares consultancies for HAL Allergy BV, Citeq BV and Angany Inc, speaker´s fees from HAL Allergy BV, ALK and ThermoFisher Scientific as well as stock options from Angany Inc, all outside the submitted work.

The remaining authors declare that the research was conducted in the absence of any commercial or financial relationships that could be construed as a potential conflict of interest.

## Publisher’s Note

All claims expressed in this article are solely those of the authors and do not necessarily represent those of their affiliated organizations, or those of the publisher, the editors and the reviewers. Any product that may be evaluated in this article, or claim that may be made by its manufacturer, is not guaranteed or endorsed by the publisher.
