# Supplementary material for: The History, Present and Future of Allergen Standardization in the United States and Europe
Source: Front Immunol. 2021 Sep 14;12:725831. doi: 10.3389/fimmu.2021.725831 (PMC8477030; doi:10.3389/fimmu.2021.725831)
Supplement: Supplementary file 1 [file DataSheet_1.docx]

1. **Supplemental Data: ID50EAL and parallel line bioassay methods**

   In the ID_50_EAL method, allergenic extracts are evaluated in subjects maximally reactive to the respective reference concentrates. Each subject is tested with serial 3-fold dilutions of the reference extract. After 15 minutes, the sum of the longest and midpoint orthogonal diameters of erythema (ΣE) is determined at each dilution, and the log dose producing a 50 mm ΣE response (D_50_) is calculated (111). Extracts that produce similar D50 responses are considered bioequivalent and are assigned similar units, the bioequivalent allergy unit (BAU). Because the modal D_50_ of a series of extracts was 14 (a 3-14 or 1:4.8 million dilution), extracts with a mean D_50_ of 14 were arbitrarily assigned the value of 100,000 BAU/mL (112). Thus, the formula for the determination of potency from the D_50_ is:
2. Potency = 3 -(14- mean D_50_) * 100,000 BAU/mL (19)(20).
3. By a similar technique and analysis, bioequivalent doses of test extracts from the same source as the reference extract can be determined by the parallel-line bioassay (39). The inverse ratio of the doses of test extract required to produce identical D_50_ responses to a reference extract is the relative potency (RP) of that extract. This analysis requires that the log dose-response curves of the test extract and the reference extract are parallel. If the two dose-response lines are not parallel, then the ratio of skin test doses for identical responses – and the RP - will vary with the dose. Because the distance between the two lines is different at each dose and a meaningful RP cannot be determined (Figure 1) (39, 112). This situation strongly suggests that there are compositional differences between the two extracts.
   In the original protocol, the D_50_ for the extract was determined by the mean D_50_ from 15 highly allergic individuals. To test the arbitrary choice of a sample size of 15, Rabin et al. (113) applied the following formula for the number of study subjects, n, that would be required:


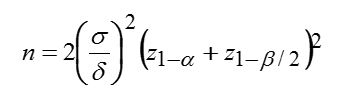


where σ is the standard deviation of the measurement, δ is the acceptable difference in D_50_s of two equivalent products, and the z values are the critical values from the cumulative normal distribution table for a significance level α and a power of 1 - β (114). From this formula, *n* is a function of the *squares* of σ and δ. The value of *n* depends on the particular allergen to be tested but, as may be seen in sample calculations represented in Table 2, *n*, the sample size to determine the D_50_ of an allergenic product, usually must be larger than 15 subjects.
